# Is an “Epigenetic Diet” for Migraines Justified? The Case of Folate and DNA Methylation

**DOI:** 10.3390/nu11112763

**Published:** 2019-11-14

**Authors:** Michal Fila, Cezary Chojnacki, Jan Chojnacki, Janusz Blasiak

**Affiliations:** 1 Department of Neurology, Polish Mother Memorial Hospital, Research Institute, 93-338 Lodz, Poland; michalfila@poczta.onet.pl; 2 Department of Clinical Nutrition and Gastroenterological Diagnostics, Medical University of Lodz, 90-647 Lodz, Poland; cezary.chojnacki@umed.lodz.pl (C.C.); jan.chojnacki@umed.lodz.pl (J.C.); 3 Department of Molecular Genetics, Faculty of Biology and Environmental Protection, University of Lodz, 90-236 Lodz, Poland

**Keywords:** epigenetic diet, DNA methylation, folate, migraine, valproic acid

## Abstract

Migraines are a common disease with limited treatment options and some dietary factors are recognized to trigger headaches. Although migraine pathogenesis is not completely known, aberrant DNA methylation has been reported to be associated with its occurrence. Folate, an essential micronutrient involved in one-carbon metabolism and DNA methylation, was shown to have beneficial effects on migraines. Moreover, the variability of the methylenetetrahydrofolate reductase gene, important in both folate metabolism and migraine pathogenesis, modulates the beneficial effects of folate for migraines. Therefore, migraine could be targeted by a folate-rich, DNA methylation-directed diet, but there are no data showing that beneficial effects of folate consumption result from its epigenetic action. Furthermore, contrary to epigenetic drugs, epigenetic diets contain many compounds, some yet unidentified, with poorly known or completely unknown potential to interfere with the epigenetic action of the main dietary components. The application of epigenetic diets for migraines and other diseases requires its personalization to the epigenetic profile of a patient, which is largely unknown. Results obtained so far do not warrant the recommendation of any epigenetic diet as effective in migraine prevention and therapy. Further studies including a folate-rich diet fortified with valproic acid, another modifier of epigenetic profile effective in migraine prophylaxis, may help to clarify this issue.

## 1. Introduction

The role of diet in the prevention and treatment of human disorders is still controversial, but many dietary compounds that can contribute to disease pathogenesis have been identified [1]. It is estimated that most human cancers in the USA are caused by external factors and diet (excluding alcohol and food additives) is the main causal external factor responsible for about 35% of cancer-related deaths [2]. Some “familial cancers” are, in fact, not attributed to the same genetic constitution of family members, but rather their similar dietary habits. Therefore, diet should be considered as a possible factor or confounder in the pathogenesis of many diseases. This raises the question of whether diet modification can be important in the prevention and therapy of diseases, not only by the avoidance of dietary elements with recognized detrimental roles in pathophysiology (e.g., an elimination diet), but also by the addition of compounds with specific mechanisms of action. 

Headache disorders including migraines, seem to be especially prone to diet as it is generally believed that some dietary ingredients and additives may trigger headache attacks [3,4]. Therefore, using an elimination diet to avoid a migraine trigger may be effective in migraine prevention, but few rigorous studies on the role of diet in headache prevention have been performed and most of them lack appropriate controls (reviewed in [5]). On the other hand, a more comprehensive diet containing specific ingredients can prevent headaches, but this is even more controversial and less studied than the elimination diet (reviewed in [6]).

Many kinds of diet are recommended to reduce risk and attenuate the detrimental syndromes of many diseases. Some diets target specific organs and some address mental and physical well-being [7]. A Mediterranean-style diet and ketogenic diet are some of the most common diets recommended to be beneficial for many human disorders [8,9,10]. However, there are no solid reports on the effect of the Mediterranean diet on migraines, but the ketogenic diet as well as high folate, low fat, modified Atkins, and high omega-3/low omega-6 diets have been reported to have some beneficial effects in the prevention of headaches including those occurring from migraines (reviewed in [6]).

Apart from diets composed of specific food or avoiding specific ingredients, diets targeting specific cellular structures and macromolecules such as mitochondria or DNA have been proposed [11,12]. The epigenetic diet, a term introduced by Hardy and Tollefsbol in 2011, is intended to target the cellular epigenetic profile and specifically mediate its changes induced by environmental factors [13]. The epigenetic diet is mainly considered in cancer prevention [14,15]. However, its rationale is based on the assumptions that diet can modify the cellular epigenetic profile and that changes in the epigenetic profile are important in cancer transformation. Both assumptions are true, but this does not necessarily mean that it is possible to compose a balanced diet specifically addressing a cancer-related, epigenetically aberrant element. Nutrition can change one’s epigenetic profile, but how to specifically regulate one’s profile with this diet is still open as it is also an important question for epigenetic drugs [16].

In this review, we critically assess the rationale behind the epigenetic diet and the findings associated with its application. We focus on migraine, DNA methylation, and folate, a nutrient frequently mentioned as an important element of epigenetic diets and also in migraine prevention [17,18,19,20]. An introductory section on the epigenetic regulation of gene expression with a special emphasis on DNA methylation/demethylation is also included.

## 2. Epigenetic Regulation of Gene Expression

The Human Genome Project has provided information on the sequence of our genome and mapped most of our genes, but now in the post-genomic era, studies aim to understand how information contained in the gene sequence is turned into a phenotype. As all our nucleated cells contain essentially the same DNA, the control of its expression in different tissues is critical for the development and functioning of our body, and deviation from it may result in disorders including serious diseases. Maintaining cellular identity and function is mostly executed by epigenetic mechanisms that include covalent modifications of genes and associated proteins and do not include changes in DNA sequences. These modifications include DNA methylation/demethylation, post-translational histone modifications, and changes mediated by non-coding RNAs (ncRNAs). The epigenome can be understood as the complement of chemical compounds that modify the expression and function of the genome [21] This complement is referred to as the cellular epigenetic profile and its changes can be considered as epimutations. 

### 2.1. DNA Methylation and Demethylation

**Figure 1:**
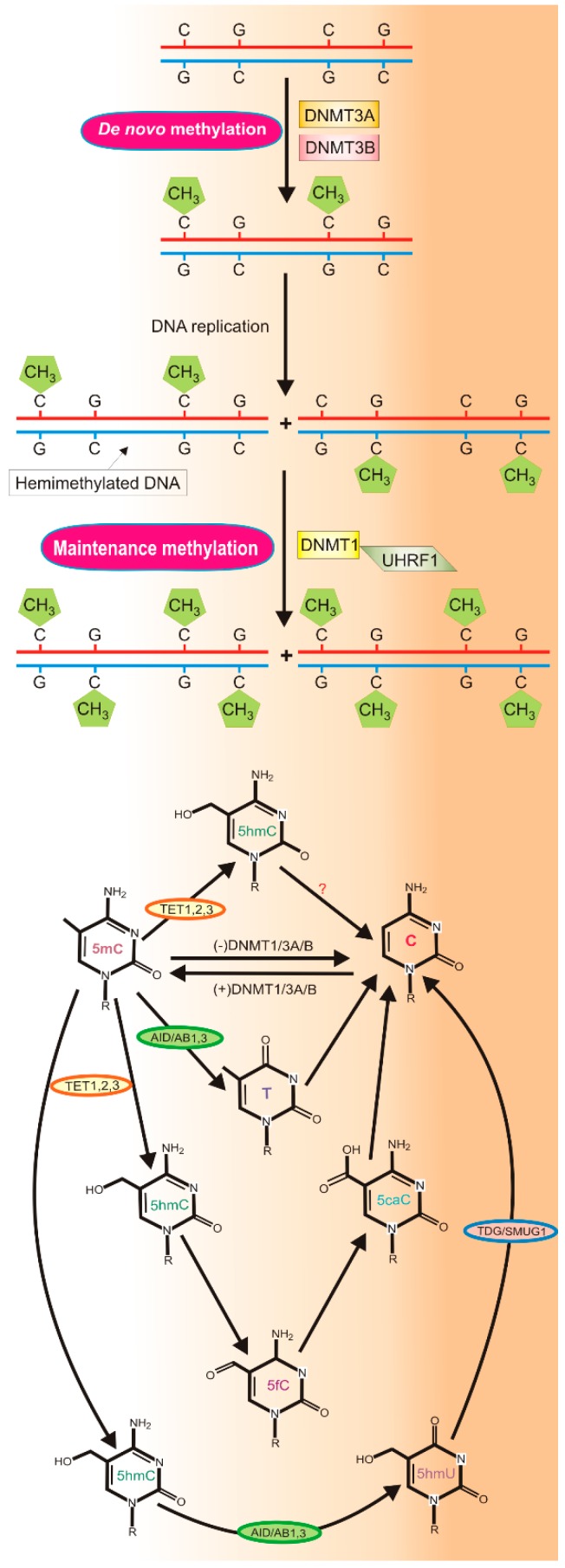
DNA methylation and demethylation in humans. DNA methylation (upper panel) producing 5-methylcytosine (5mC) is catalyzed by DNA methyltransferases (DNMT). DNMT1 methylates hemimethylated DNA (maintenance methylation) and can be assisted by UHRF1 (ubiquitin like with PHD (plant homeodomain) and ring finger domains 1). DNMT3A and DNMT3B are involved in de novo DNA methylation; 5mC can be reverted to C passively or actively (lower panel) and may undergo spontaneous or activation-induced deaminase (AID)-mediated deamination converting it into thymine (T), which can be replaced with C via DNA repair with TDG (thymine DNA glycosylase) or SMUG1 (single-strand-selective monofunctional uracil-DNA glycosylase 1) glycosylases. Active demethylation is led by the ten eleven translocation (TET) proteins TET1-3 with the following intermediates: 5-hydroxymethylcytosine (5hmC), 5-formylcytosine (5fC), and 5-carboxylcytosine (5caC). 5hmC can be converted to 5 hydroxymethyluracil (5hmU) or T.

The process of DNA methylation involves the transfer of a methyl group from S-adenosyl-methionine (SAM) to the carbon-5 of cytosine (C) residue in DNA, resulting in its change to 5-methyl cytosine (5mC). SAM is produced in one-carbon metabolism centered at folate and methionine cycles (Figure 1). In humans, this process is catalyzed by the DNA methyltransferases DNMT1, DNMT3A, and DNMT3B [22]. 

DNMT1 is involved in DNA maintenance methylation and methylates hemimethylated DNA on its non-methylated strand. It is recruited to DNA through its interaction with UHRF1 (ubiquitin like with PHD and ring finger domains 1) [23] (Figure 1). DNMT3A and DNMT3B are de novo methyltransferases that methylate unmethylated DNA on both strands. DNMT3A can be assisted by the catalytically inactive regulatory factor DNMT3L (DNA methyltransferase 3 like) [24]. Apart from DNMT1/3A/3B, another methyltransferase, DNMT2, occurs in humans [25]. This enzyme is involved in cytosine RNA methylation, but not all its functions are known.

The methylation of C is common in the human genome and involves mainly cytosine within a 5-’CpG-3′ (CpG) dinucleotide; a genome-wide high-resolution analysis in human fibroblasts revealed that 99.98% of DNA methylation occurred in CpG dinucleotides [26]. Certain regions of the human genome have more than ten-fold higher frequency of CpG occurrence than the rest of the genome and are called CpG islands. These islands are frequently associated with gene regulatory regions including promoters, and are usually unmethylated (reviewed in [27]). CpG islands have “shores”, which are 2 kb regions containing CpG dinucleotides at a low frequency and are involved in tissue-specific methylation [28].

5mC can be reverted to C passively or actively (Figure 1) and may undergo spontaneous or activation-induced deaminase (AID)-mediated deamination, thereby converting it into thymine (T) that can be replaced with unmodified cytosine by G/T mismatch-specific thymine DNA glycosylase (TDG) or the canonical mismatch repair system (MMR). Active DNA demethylation occurs with the involvement of the ten eleven translocation (TET) proteins TET1, TET2, and TET3 [29]. This process proceeds through TET2-mediated oxidation of 5mC to 5-hydroxymethylcytosine (5hmC), 5-formylcytosine (5fC), and 5-carboxylcytosine (5caC). Oxidized 5mC is progressively lost in subsequent cellular divisions or converted to non-methylated C by TDG that can also remove 5fC and 5caC. AID converts 5hmC to 5 hydroxymethyluracil (5hmU) or T. All these modifications of C can be processed by TDG or other glycosylases of base excision repair or MMR.

DNA methylation/demethylation regulates gene expression by various, not completely known, mechanisms. Usually, DNA methylation of the CpG islands, especially those located in the regulatory regions, results in gene silencing. Methyl groups in the major groove of the DNA may sterically constrain the binding of transcription factors. Methylated CpG dinucleotides may be targeted by specific proteins recognizing such modified dinucleotides and binding DNA in the regions in which they occur, preventing the access of transcription factors. 

DNA methylation is required for normal brain development and its disturbance is causatively associated with Rett syndrome, an autism spectrum disorder, as well as fragile X syndrome [30,31,32]. DNA methylation plays a role in the diverse functions of the brain including neuronal activity, learning and memory, degeneration, and brain addiction [33].

### 2.2. Histone Modifications and Non-Coding RNAs

The human genome is packaged in a highly organized structure, chromatin, whose major components are histones. These histones are subjected to an array of post-translational covalent modifications, especially in their N-terminal tails, which protrude from histone complexes and are accessible to histone-modifying enzymes. These modifications are an important part of the genetic information carried by the fragment of DNA associated with histones and this is why they are referred to as the histone code [34]. The histone code is an important, epigenetic element in the regulation of gene expression as it determines the accessibility of a DNA fragment for the RNAs and proteins of transcriptional machinery by signaling proteins to remodel the structure of chromatin. The histone code, which is a combination of various chemical modifications of histone tails including acetylation, methylation, phosphorylation, ubiquitination, sumoylation, and others, is established post-translationally by the histone-modifying enzymes that form large protein complexes with DNA-binding proteins and chromatin remodeling enzymes [35]. These complexes regulate DNA replication, transcription, and repair as well as other cellular processes in an epigenetic fashion [36]. 

Regulatory non-coding ncRNAs can be divided into two classes: short RNAs (sRNAs) and long ncRNAs (lncRNAs). sRNAs typically have tens to a few hundreds of nucleotides and include micro RNAs (miRNAs), small interfering RNAs (siRNAs), Piwi-interacting RNAs (piRNAs), and repeat-associated small interfering RNAs (rasiRNAs) that regulate gene expression through RNA interference by base-pairing with their targets. LncRNAs are transcribed from non-protein-coding regions of the genome and range from thousands to hundreds of thousands nucleotides long and their characterization is an emerging area of research [37]. LncRNAs may use different mechanisms to regulate gene expression. For example, probably the most well-known lncRNA, the Xist RNA, inactivates the X chromosome by coating one X chromosome and recruiting proteins to inactivate it [38,39].

In summary, the epigenetic profile is dynamically regulated by several objects that interact with each other. Not all of these interactions are known or can be predicted, which is why any epigenetic intervention can be associated with a relatively high degree of uncertainty.

## 3. The Epigenetic Diet—Does It Really Exist?

Similar to the genome, the epigenome can be modified by internal and external factors including components of the diet. 

Changes to the genome sequence induced by diet are initiated by DNA modifications that are targeted by the cellular DNA damage response (DDR). A substantial difference between genetic and epigenetic modifications by dietary compounds is that the latter can be changed with much higher probability than the former. Therefore, diet seems to be a more convenient tool to modify the epigenome than genome. However, epigenetic modifications are more difficult to control as (I) the histone code is not fully known; (II) the number of possible combinations of chemical modifications of all histone tails is enormously high; and (III) histone modifications are combined with the DNA methylation/demethylation status and action of ncRNAs to produce the epigenetic profile. Nevertheless, many drugs targeting proteins responsible for cellular epigenetic pattern have been approved in cancer therapy or have been investigated in clinical trials (reviewed in [40]). This class of treatment with drugs targeting the epigenetic profile is known as epigenetic therapy. Epigenetic drugs mainly target DNA methylation and the chemical modifications of histones (reviewed in [41]).

Chemical compounds including diet components or supplements can change both the genome and the epigenome. One of the most direct pieces of evidence showed that the methyl-deficient—lacking sufficient levels of folate, choline and methionine—induced abnormal DNA methylation in the liver of mice that developed methyl-deficiency-induced hepatocarcinoma [42]. 

**Figure 2:**
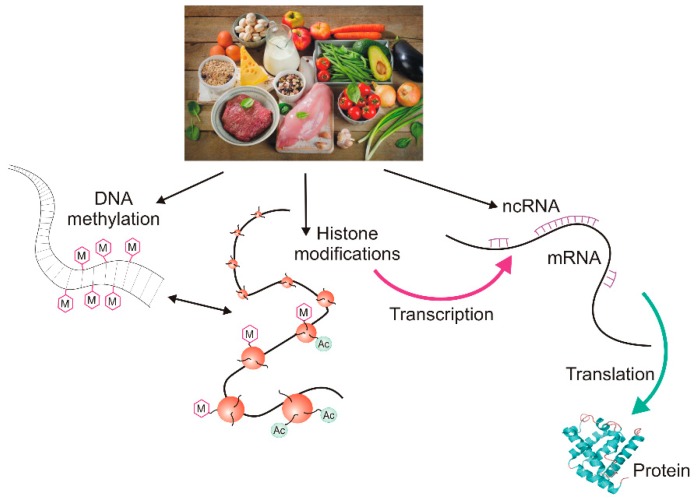
The epigenetic diet. Dietary compounds can affect all main elements of the cellular epigenetic profile: DNA methylation, histone modification, and the action of non-coding RNAs (ncRNAs) resulting in changes in transcription and/or translation and finally, the ultimate product of gene expression: protein or RNA. M denotes a methyl group and Ac denotes an acetyl group. The dietary products presented in the picture symbolically represent the epigenetic diet and do not necessarily reflect their actual ability to modify the epigenetic profile.

Chemicals can change the epigenetic profile directly or indirectly through interactions with epigenetic factors (i.e., chemicals, proteins or RNAs involved in establishing the epigenetic profile). These chemicals can be components of a regular or special epigenetic diet, and, in fact, it is difficult to find a diet that does not have any potential to change the epigenetic profile (Figure 2). However, there may be a substantial difference between the action of a chemical administrated alone and when it is given as a food component. This difference may be underlined by the different bioavailability of this chemical. The bioavailability of a single product may differ from its bioavailability when it is administrated along with other substances that may change its action. Therefore, any natural dietary product or diet supplement should be considered with other substances that may act synergistically, increasing or decreasing its bioavailability and final biological effects. For instance, the bioavailability of vitamin C, either natural or synthetic, can be modulated by dietary flavonoids. Dehydroascorbic acid (DHA), the main oxidized form of vitamin C, is transferred by glucose transporters, so it must compete with glucose, which is present in many natural sources of vitamin C. Moreover, some flavonoids, plant-derived health-beneficiary substances, have been reported to inhibit vitamin C and DHA transporters [43,44,45,46]. However, many flavonoids display antioxidant properties and their action can spare the pool of vitamin C molecules that would otherwise be oxidized [47]. Therefore, consequences of epigenetic diet may be much more difficult to predict than the effects of epigenetic drugs. In addition, tissue-specific absorption may be different for a substance acting alone and in combination with other substances. Moreover, the epigenetic diet, like any other kind of diet, requires personalization due to the individual features of the recipient, but our epigenetic profile is relatively poorly known and has a dynamic character as it must react to changes in environmental conditions and the needs of the organism resulting from its development. Finally, as will be shown in the next sections, the effects of diet on migraines may depend on gender.

The emerging role of epigenetic drugs in cancer therapy is associated with two opposite effects: global DNA hypomethylation and the local hypermethylation of the promoters of genes involved in maintaining genome stability including tumor suppressors [48]. Global hypomethylation can include promotors of oncogenes and lead to their activation. DNA methylation-oriented epigenetic drugs can induce global changes in the methylation pattern, which can also include oncogenes. However, no epigenetic drug is currently known to act specifically on oncogenes or other cancer-related genes, and, consequently, changes in the epigenetic profiles of other genes are expected via the action of that drug. This may lead to the inactivation of genes that act as tumor suppressors and other genes important for cellular homeostasis. It can be expected that the degree of uncertainty about the final effect would be even higher when a bioactive dietary compound instead of a clinically tested drug is administrated. Therefore, the incorporation of a bioactive compound of an epigenetic diet into a balanced regular diet to induce the required changes in epigenetic patterns still needs further study.

In summary, the use of the term “epigenetic diet” is, at present, not fully justified and should not be understood like other relatively well established kinds of the diets including the Mediterranean diet or ketogenic diet.

## 4. Migraine and Diet

Migraines are a common (2013 estimated global prevalence 14.7%) brain disorder with severe headaches and associated neurological and systemic symptoms [49]. Based on the frequency of occurrence, the International Headache Society classifies migraines as episodic or chronic migraines. Migraines may occur in two main clinical subtypes: migraine with aura (MA) and without aura (MO). A migraine aura may precede a headache attack, occur during the attack, or appear without a headache [50]. 

**Figure 3:**
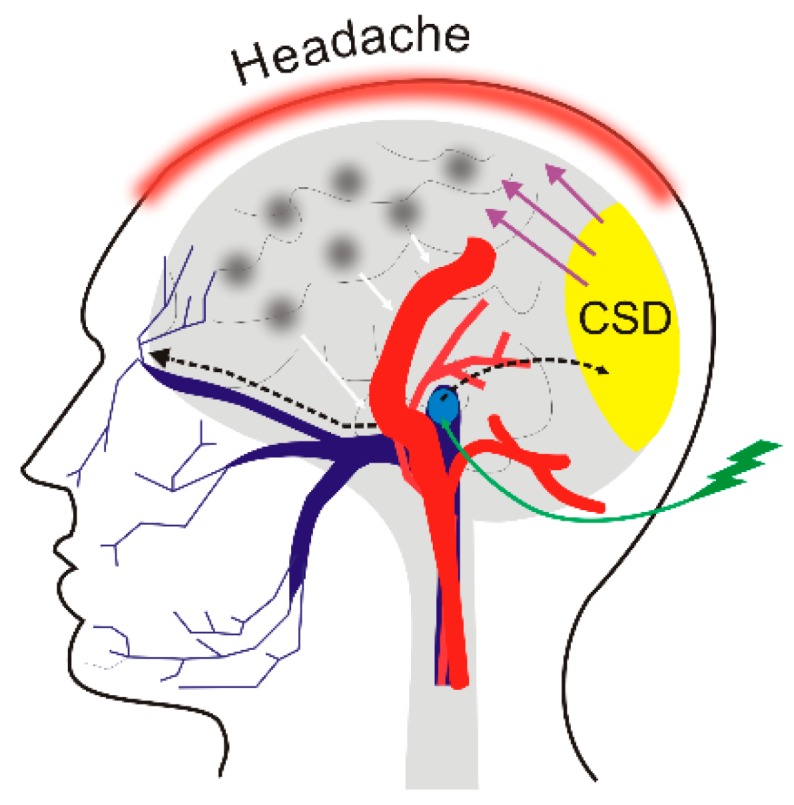
A putative mechanism for migraine headaches induced by a trigger. A migraine trigger (green thunder) affects the nucleus (light blue oval) of the trigeminal nerve (dark blue) and activates it. This results in waves of depolarization (black broken arrows) moving along the nerve and reaching the cortex and evoking cortical spreading depression (CSD). This results in neurogenic inflammation (black clouds) and release of inflammatory neurotransmitters (white arrows), which induce dilation of the brain blood vessels (red), which causes the release of pain-producing prostaglandins that in turn evoke a migraine headache. The specific order of events presented here is hypothetical and requires validation.

The aura may include several visual and mental syndromes and is believed to relate to cortical spreading depression (CSD), an important effect in migraine. However, the exact mechanism whereby CSD is initiated is not exactly known, nor is it known how CSD initiates the subsequent phases of migraine. Some possible mechanisms including the activation of the trigeminal nerve and the induction of neurogenic inflammation are presented in Figure 3 [51]. A trigger may be an environmental or lifestyle factor such as stress, a light flash, physical effort, noise, sleep disturbance, or diet [52]. 

Although the prevention and treatment of migraines remain challenging [53], migraine drugs approved by the FDA have lately produced hope for a breakthrough [54] These drugs include erenumab (Aimovig), fremanezumab (Ajovy), and galcanezumab (Emgality), which are all monoclonal antibodies and GRPC antagonists. It is too early to draw a definite conclusion on the general role these drugs may play in migraine treatment, but the first observations are prospective, despite the relatively high cost of therapy with these drugs, estimated at about $575 per month [55].

**Figure 4:**
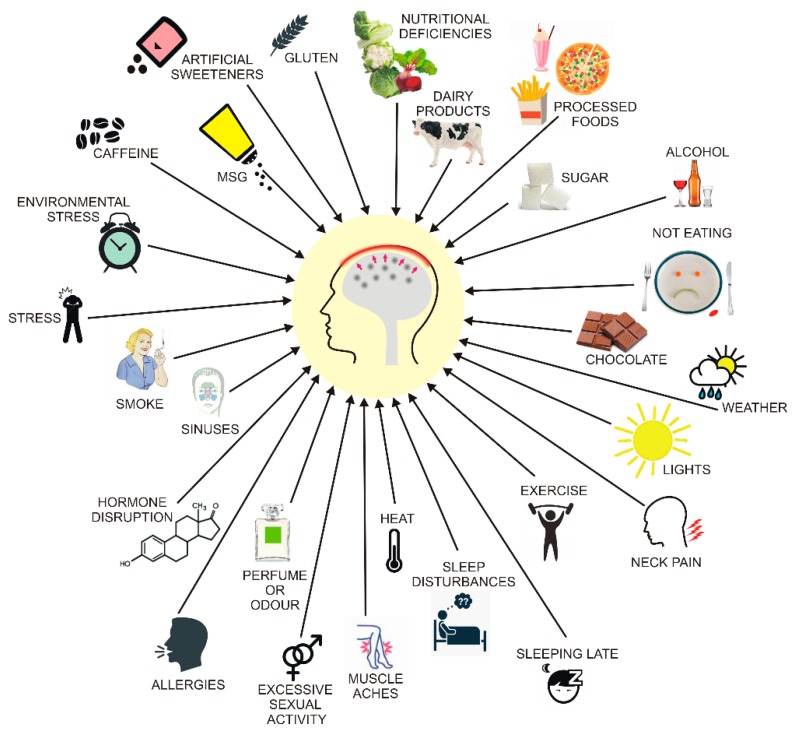
Main migraine triggers. Some are well established and confirmed by reports on large cohorts, but others are problematic and require further research. Recent research suggests that some food triggers are actually food cravings experienced as the first phase of migraine before pain onset. Their action and threshold can be modulated by several environmental and genetic factors that act synergistically.

The pathogenesis of migraine is largely unknown, but both genetic and environmental factors may be involved. These factors can modulate the threshold for a migraine trigger that precedes and evokes a migraine attack [56]. Many potential migraine triggers have been identified and a substantial fraction of them is associated with food (Figure 4). Female sex hormones, the menstrual cycle, and pregnancy modulate migraine attacks, so they may contribute to the approximately three times higher prevalence of migraines in women than in men [57]. This relationship may also be underlined by the X-linked form of this disease or the mitochondrial transmission of its other form or both [58]. 

The genetic basis of migraine is supported by the association of migraine with mutations in a single gene (monogenic migraine) or clusters of genes (polygenic migraine) (reviewed in [59]). Mutations in the three ion channels genes, *CACNA1A* (calcium voltage-gated channel subunit alpha 1 A), *ATP1A2* (ATPase Na^+^/K^+^ transporting subunit alpha 2), and *SCN1A* (sodium voltage-gated channel alpha subunit 1) were identified as specifically causal for hemiplegic migraines, a rare variant of MA, and genome-wide association studies have identified 38 loci associated with increased risk of migraines [60]. Many other genes are candidates of importance in migraine pathogenesis, but a substantial majority of them have not been convincingly replicated [61]. However, these are not genes themselves, but their expression directly determines the migraine’s phenotype. As mentioned, the cellular epigenetic profile is an important element of the regulation of gene expression. Epigenetics is also a significant element of pathogenesis in many human diseases including behavioral and brain disorders [62]. Several chemicals targeting the epigenome have been accepted as drugs or are under clinical trials [63]. Valproic acid (VPA), a histone modifier, has been applied for more than 50 years in epilepsy treatment and is currently used in the therapy of bipolar disease and the prophylaxis of migraines [64,65]. The role of epigenetic modifications in migraine is not completely known, but epigenetics is considered to be a promising avenue in the prophylactic treatment of this disease [66].

The cellular epigenetic profile is more prone to nutritional modifications than corresponding DNA sequence [67]. Therefore, epigenetically active nutrients can affect the pathogenesis of human disorders and nutriepigenomics is also a promising avenue in the prevention and therapy of human complex diseases [1]. This issue seems to be especially important in migraines, as it is frequently related to improper diet, and the avoidance of certain nutrients in the diet is an important element of its prophylaxis and often results in a decreased severity of headaches [68]. Much less is known about the prevention of migraine and the attenuation of its symptoms via the active supplementation of the diet. The ketogenic diet is considered to be a rapid onset effective prophylaxis for episodic and chronic migraines, and ketosis was recently suggested to regulate cellular functions through interactions with the epigenome, but our knowledge of the mechanisms of this interaction is far from complete [69,70]. However, a ketogenic diet is not the only diet that may affect the epigenome as many compounds not included in such a diet are reported to do so [13].

In a large (8042 men and 23,728 women) cross-sectional study on subjects from a population-based NutriNet-Santé e-cohort, Andreeva et al. observed migraine occurrence in 9.2% of men and 25% of women [71]. They also observed lower protein and higher fat consumption in male migraineurs than in males without headaches and those with non-migraine headaches and higher fat and carbohydrate intake in female migraineurs than females without headaches and those with non-migraine headaches. These results indicate a gender-specific difference in the consumption of macronutrients among migraineurs. However, whether this difference contributes to different prevalence of migraine between men and women should be confirmed by further research, as the differences observed in these large cross-sectional studies were not very pronounced. These and other studies show that nutrition may be important in migraine pathogenesis, and this problem should be considered along with other genetic and environmental migraine-related factors. Further details on the role of diet in migraine pathogenesis are provided in the next sections.

In summary, the use of the term “epigenetic diet” is, at present, not fully justified and should not be understood in a similar way to other relatively well established kinds of diets including the Mediterranean diet or ketogenic diet.

## 5. DNA Methylation in Migraine

A migraine trigger must reach a threshold to induce headaches and this threshold may be lowered by frequent headache attacks through epigenetic mechanisms [66].

In a recent 11-year retrospective case-control study, Winsvold et al. showed that the transformation from episodic to chronic headaches in mixed headache and migraine patients was associated with changes in the DNA methylation profile compared to the headache-free controls [72]. DNA methylation was assessed in 485,000 CpG sites at two stages and a combined meta-analysis revealed that the strongest associated CpG sites were related to the *SH2D5* (SH2 domain containing 5) gene, whose product may be involved in the regulation of synaptic plasticity through the control of Rac-GTP levels and NPTX2 (neuronal pentraxin 2), which is involved in the inhibition of excitatory synapse formation through the binding and clustering of the glutamatergic AMPA (α-amino-3-hydroxy-5-methyl-4-isoxazolepropionic acid) receptor. Therefore, both genes are implicated in the regulation of synaptic plasticity playing a role in migraine pathogenesis [72]. Functional analysis suggests the involvement of the calcium ion binding and estrogen receptor pathways—both strongly associated with migraine pathogenesis. This first genome-wide study showed that elements of migraine pathophysiology might be epigenetically changed during its chronification from episodic to chronic form. However, neither SH2D5, nor NPTX2, have been reported to be involved in migraine pathogenesis.

Terlizzi et al. looked for changes in the DNA methylation profile associated with headache chronification compared with the controls without headache (HC), episodic migraineurs (EM), and patients with chronic migraine with medication overuse headache (MOH) in a 6-month follow up study [73]. In this pilot study, which was performed on a small number of patients, no difference was found between the MOH and EM groups, but several genes were identified to change their DNA methylation profile in the chronification in MOH as compared with the controls including *COMT* (catechol-O-methyltransferase), *ZNF234* (zinc finger protein 234), and *SOCS1* (suppressor of cytokine signaling 1), which are all involved in the pathogenesis of drug addiction and neuropsychiatric illness. Variability in the *COMT* gene was associated with migraine, but the two remaining genes did not relate to migraine [74,75]. No difference was found between the MOH and EM groups. These results require confirmation in larger samples.

In another epigenome-wide association study performed on the blood of 67 migraineurs and 67 controls, Gerring et al. identified 63 differentially methylated regions (DMRs) rich in regulatory elements close to genes whose products are involved in solute transport: *SLC2A9* (solute carrier family 2 member 9), *SLC38A4, SLC6A5* and cellular homeostasis: *DGKG* (diacylglycerol kinase gamma), *KIF26A* (kinesin family member 26A), DOCK6 (dedicator of cytokinesis 6), and *CFD* (complement factor D) [76]. As many drugs can influence the epigenetic profile this study suffers from the drawback that no medication information was collected.

Wan et al. presented rather weak evidence for an association between low levels of the methylation of the RAMP1 (receptor activity modifying protein 1) gene in blood and a higher migraine risk in females [77]. The RAMP1 protein is a key receptor subunit of the calcitonin gene related peptide (CGRP), and both are expressed in trigeminal neurons and are essential for migraine pathogenesis [78]. The CGRP gene is normally inactive in trigeminal glia, but it was shown that epigenetic modifications resulted in its activation in rat cells in vitro [79]. Whether or not this observation is related to migraine pathogenesis should be further established as there are several questions associated with that study (for example, can an in vitro study on rat cells be associated with human migraines?). However, these studies were performed on peripheral blood leukocytes, but DNA methylation is tissue-specific as it is involved in tissue-specific gene expression.

Labruijere et al. showed that the methylation of genes associated with migraines—*CRCP* (CGRP receptor component), *CALCRL* (calcitonin receptor like receptor), *ESR1* (estrogen receptor 1), and *NOS3* (nitric oxide synthase 3)—was tissue-specific in female rats and the methylation of these genes in leukocytes did not correlate to methylation in other tissues [80]. On the other hand, such methylation correlated well with the methylation of the corresponding genes in human leukocytes, and the authors postulated that rats represent a good model for the study of DNA methylation in human materials that are difficult to obtain.

In summary, several genes have been reported to change their methylation profile with migraine occurrence or progression, and some of them were previously associated with migraine pathogenesis (Table 1).

## 6. Folate and Its Role in DNA Methylation and Migraine Pathogenesis 

**Figure 5:**
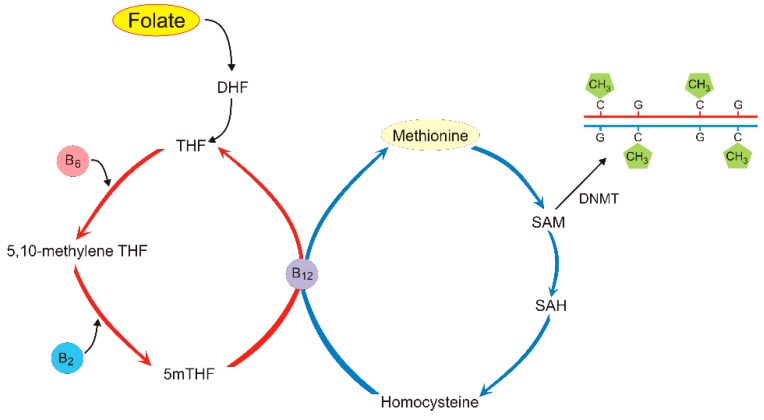
DNA methylation in one-carbon metabolism centered around the folate (**left**) and methionine (**right**) cycles. Folate is reduced to dihydrofolate (DHF) and tetrahydrofolate (THF). THF is changed into 5,10-methylene THF with the possible involvement of vitamin B6; 5,10-methylene THF is converted to 5mTHF, which is demethylated to complete the folate cycle. Vitamin B2 can also be involved in these steps. Carbon from the 5mTHF demethylation enters the methionine cycle through the methylation of homocysteine to produce methionine by methionine synthase with vitamin B12 as a cofactor. Methionine may generate S-adenosyl-methionine (SAM), which provides methyl groups for DNA methyltransferases (DNMTs) that methylate DNA. SAM is then demethylated to S-adenosylhomocysteine (SAH), which is converted back to homocysteine.

Folate (folacin, vitamin B9) is one of the B vitamins and an essential micronutrient that plays a critical role in one-carbon cellular metabolism [81,82]. Humans, as mammals, cannot synthesize folate and must intake it with food either as a component of a natural diet, or as a fortified food or diet supplement. Folate supplementation, recommended in many countries, can come in the form of folic acid, folinic acid, or 5-methyltetrahydrofolate (5-MTHF). 5-MTHF occurs naturally and has some advantage over synthetic forms of folate including its higher bioavailability [83]. Folate is essential for many cellular effects such as nucleoside synthesis and the methylation of biomolecules including DNA (Figure 5).

Dietary folate is metabolized to 5-methyltetrahydrofolate (5mTHF, monoglutamyl form) by methylenetetrahydrofolate reductase (MTHFR). This reaction is important for the remethylation of homocysteine to methionine, which is a substrate for SAM, providing methyl groups for DNA methyltransferases to methylate DNA [84]. Several other dietary nutrients are required to maintain the one-carbon flux needed for DNA methylation including vitamins B2, B6, and B12, riboflavin, and choline (Figure 5) [85].

Low folate status is associated with an increased risk of several disorders including cardiovascular diseases (CVD) and cancer, but the mechanisms underlying these associations are not exactly known, and several pathways may be involved [86,87]. However, the results of some folate intervention trials suggest that excessively high folate supplementation may be detrimental for a person with an elevated risk of cancer and CVD (reviewed in [88]). That review summarized studies with the supplementation of both folate and folic acid, which were not adequate due to inter-individual variability in the activity of the 5,10-methylene THF reductase. Therefore, the dose-effect relationship for folate in CVD may be nonlinear.

Folate deficiency could be also involved in disorders of the nervous system [89,90]. Folate is an important factor in the functioning of the blood–brain barrier and brain development [91]. Variability of the *MTHFR* gene could result in phenotypic differences: the T allele of the 677C > T polymorphism of this gene is associated with elevated levels of plasma homocysteine [92]. An excess of homocysteine can be detrimental for vessels and result in endothelial cell injury and changes in blood properties that can be important in CVD and migraine pathogenesis [93,94]. 

Novel epigenomic loci associated with dietary folate and vitamin B12 intake were identified in a large-scale epigenome-wide association study on 5841 individuals [20]. These studies identified significant differentially methylated positions (DMPs) and regions (DMRs) in the genome, and a pathway analysis was performed on DMR annotated genes. Vitamin B12 intake was associated with 29 DMRs annotated with 48 genes. Folate intake was negatively associated with six DMPs annotated with five genes involved in cellular processes including centrosome localization, cell proliferation, and tumorigenesis. In these studies, vitamin intake was assessed on the basis of a questionnaire. 

That work can be considered in the context of the study by Illingworth et al., which assayed 1.9 million CpG islands in each of the 43 brain samples and showed over 16,000 DMRs [95]. These authors concluded that except for the cerebellum, patterns of DNA methylation in different brain regions were more similar than the patterns for those regions in different individuals. Therefore, human brain methylome is primarily determined by DNA sequence and not developmental status. Although it is assumed and supported by many studies that the DNA methylation pattern is stable and retains in isolated genomic DNA, it is not completely known as to which changes in the epigenome are associated with death. 

The 677C > T polymorphism of the *MTHFR* gene is likely the most frequently addressed genetic aspect of migraine pathophysiology, but the results obtained so far are not conclusive [96]. This polymorphism is claimed to be both an independent and combined marker for migraines, especially MA. Several meta-analyses addressing this polymorphism in migraines have been performed. Liu et al. concluded that the 677T allele was associated with an increased risk of total migraine and MA in Asians [97]. Similar results were obtained in other analyses with the general conclusions supporting the use of folate in migraine patients, especially those with auras, but further replication studies are needed, particularly large randomized clinical trials [96].

Menon et al. observed an inverted relationship between folic acid consumption and migraine frequency in 141 females [19]. This relationship was modulated by the 677C > T polymorphism of the *MTHFR* gene. Similar effects were noted in children with migraines and hyperhomocysteinemia [98]. Vitamin supplementation including 2 mg/day of folic acid reduced the prevalence of MA disability from 60% to 30% after six months [99]. A randomized, double-bind, placebo-controlled study (*n* = 95) showed that folic acid at 5 mg and vitamin B6 at 80 mg decreased headache frequency and headache severity [100]. 

A case-control study performed on 124 migraine patients and 130 non-migraine subjects revealed a lower level of dietary folate intake in migraineurs [18].

No association was found between the 134R > K and 653R > Q polymorphisms of the *MTHFD1* (methylenetetrahydrofolate dehydrogenase, cyclohydrolase, and formyltetrahydrofolate synthetase 1) gene, whose product is important in folate metabolism and migraine occurrence in 162 MO and 358 MA Australian patients [101]. Moreover, these two polymorphisms did not change the increased migraine risk associated with the 677T allele of the *MTHFR* gene.

Although the exact mechanism connecting the 677C > T polymorphism of the *MTHFR* gene with migraine pathophysiology is not completely known, some pathways can be considered. The C→T transition at 677 leads to the substitution of alanine to valine, thereby resulting in reduced activity of the MTHFR enzyme compared to its wild-type counterpart [102]. Consequently, individuals homozygous for the T variant have higher homocysteine levels than the C homozygotes [103]. As stated previously, an excess of homocysteine can be destructive for vessels and play a role in migraine pathogenesis, especially in migraine with an aura [104]. However, the direct link between homocysteine level and migraines is still a matter of debate, especially since only one study so far has evaluated the level of homocysteine in the cerebral fluid of migraineurs [105]. Nevertheless, elevated homocysteine may cause injury to endothelial cells, reduced flexibility of the vessels, and changes in hemostasis, which may contribute to headaches and the many associated effects and even vascular comorbidity of migraines, especially MA [106]. Homocysteine and its related compounds may act as excitatory agonists of the NMDA (*N*-methyl-d-aspartate) subtype of glutamate receptors, which are important for CSD [107,108]. Other potential aspects of the significance of the 677T > C polymorphism in the *MTHFR* gene such as its association with calcitonin gene related peptide or migraine triggers have not been investigated so far and require further research. Several cross-sectional, prospective, or interventional studies suggest that elevated plasma levels of homocysteine are associated with an increased risk of migraines (reviewed in [17]). The production of homocysteine requires folate and vitamins B6 and B12 whose deficiency results in DNA hypomethylation, which was hypothesized to trigger migraine resulting from an interplay with MTHFR and variants of estrogen receptor 1 [109].

In summary, folate is essential for DNA methylation and its presence in the diet was reported to exert a beneficial effect on migraines. However, these profitable effects of dietary folate have not been attributed to changes in DNA methylation or other alterations in the epigenetic profile.

## 7. Conclusions and Perspectives

Dietary intervention in a disease is always attractive as it is rarely associated with serious side effects. However, even a simple diet contains many components that may interact with many genes in many ways. This may lead to effects that are difficult to predict. On the other hand, attempts to isolate the impact of targeted dietary modifications may make little, if any, sense, as they might lead to the replacement of a diet with a drug. Therefore, planning therapy with dietary intervention is a risky task. An avoidance diet, eliminating compounds that are known to exert detrimental effects, is usually easier to apply than comprehensive diet, containing specific compounds that exert beneficial effects. However, a long-term elimination diet can result in undernutrition, a form of malnutrition, which is characterized as the inadequate intake of protein, energy, and micronutrients, and may result in disorders including psychological loads or infection [110]. An easy and quick tool to evaluate the risk of malnutrition resulting from an elimination diet is the Malnutrition Universal Screening Tool (MUST), which can be used to determine nutritional risk [111]. 

When considering the application of an epigenetic diet for migraines, we should determine its advantages over epigenetic drugs that can be administrated in a more controlled way with a potentially more specific action. Our answer is: there are no advantages. The only possible advantage is that a drug with known epigenetic action may not act effectively on migraines when administrated alone, but it may prove to be efficient when given in a combination with dietary components. However, this is rather illusory advantage as similar and ineffective, but generally neutral, effect of a drug may change into an adverse one when added as a diet supplement.

As many dietary factors are known to be putative migraine triggers, an avoidance diet may be effective in migraine prophylaxis and treatment. However, dietary intervention is not specific for migraines as many other diseases include diet in their pathogenesis and no evidence for an epigenetic mechanism underlying the efficacy of an elimination diet has been reported. 

We have presented results of studies showing that the methylation of some genes including those with likely involvement in migraine pathogenesis may be different for migraines. On the other hand, the variability of some genes involved in folate metabolism has been reported to correlate with migraine occurrence. Do these results together allow us to draw the conclusion that a diet rich in folate may be effective in migraine through the modification of the DNA methylation pattern? This conclusion is not justified by the results of the studies performed so far.

The largest proportion of studies on the role of epigenetics for migraine relate the prophylactic properties of VPA and its derivatives, but most of them do not definitely analyze changes in histone modification and/or DNA methylation [112]. The mechanism of prophylactic action of VPA in migraines is not completely known, but VPA inhibits the GABA (gamma-aminobutyric acid)-degrading enzymes aminotransferase and succinic semialdehyde, and increases GABA postsynaptic effects, thereby increasing the neuro-inhibitory activity of this neurotransmitter [113]. These effects may contribute to the inhibition of CSD. Furthermore, VPA may decrease neurogenic inflammation by decreasing the plasma extravasation of vasoactive neuropeptides [114]. However, VPA is primarily used as an anticonvulsive drug with many modes of action and many side effects and it is not known whether its prophylactic action in migraine is related to its epigenetic properties [115].

Although VPA is a histone deacetylase inhibitor, it can change the DNA methylation pattern; it may actively demethylate DNA in a replication-independent fashion [116]. Moreover, VPA demethylates specific genes, thus enhancing its therapeutic potential [117]. VPA was reported to decrease methylation levels in the promoters of specific genes (tumor suppressors), but increase overall methylation, which can be attributed to its anticancer properties [118]. VPA is used in the form of concentrated drops to fortify some diets including the ketogenic diet [119]. Therefore, if the epigenetic diet is a promising avenue for migraine treatment, studies on diets fortified with VPA should be undertaken. Notably, VPA has teratogenic potential and folate is speculated to act protectively against the side effects of VPA, although some results are contradictory [120,121,122]. 

The final DNA methylation pattern is underlined by the concerted and mutual expression of the genes involved in methylation, demethylation, and folate-mediated one-carbon metabolism pathways [123]. In this review, we showed the results of several studies and trials suggesting that folate administration may have a beneficial effect on migraineurs. However, there is not sufficient evidence to attribute this beneficial effect to the epigenetic action of folate, and further studies are needed to clarify this problem. Therefore, diets fortified with folate, which are beneficial in migraine, cannot be classified as epigenetic diets for this disease, although such diets can alter the epigenetic profile and lower the detrimental effects related to migraines. 

In conclusion, “epigenetic diet” in general is a misleading term, as it is difficult to find a diet that would not affect the epigenetic profile. The application of an epigenetic diet in the prophylaxis and treatment of diseases whose pathogeneses are related to changes in the epigenetic profile is rather elusive, as it is difficult to foresee the diet’s final effect due to low specificity of such a diet to the epigenome and the high number of interactions among the active components of the diet. There is no substantial evidence that folate-rich diets are therapeutically effective for migraines. The combined effect of folate and valproic acid in migraine may be investigated to determine dietary recommendations for this disease. Furthermore, an avoidance diet eliminating migraine triggers should be studied to determine its relationship with epigenetic events.

## Figures and Tables

**Table 1:** Genes whose methylation can be associated with migraine occurrence.

	Full Name	Reference
SH2D5	SH2 domain containing 5	[72]
COMT *	catechol-O-methyltransferase	[73]
ZNF234	zinc finger protein 234	[73]
SOCS1	suppressor of cytokine signaling 1	[73]
SLC2A9, SLC38A4, SLC6A5	solute carrier family 2,38A,6A member 9,4,5	[76]
DGKG	diacylglycerol kinase gamma	[76]
KIF26A	kinesin family member 26A	[76]
DOCK6	dedicator of cytokinesis 6	[76]
CFD	complement factor D	[76]
RAMP1 *	receptor activity modifying protein 1	[77]
CGRP *	calcitonin gene related peptide	[80]
CRCP *^1)^	CGRP receptor component	[80]
CALCRL *^1)^	calcitonin receptor like receptor	[80]
ESR1 *^1)^	estrogen receptor 1	[80]
NOS3 *^1)^	nitric oxide synthase 3	[80]

* denotes genes reported previously to associate with migraine, ^1)^ DNA methylation studied in rats.
